# From microscopy data to *in silico* environments for *in vivo*-oriented simulations

**DOI:** 10.1186/1687-4153-2012-7

**Published:** 2012-06-26

**Authors:** Noriko Hiroi1, Michael Klann, Keisuke Iba, Pablo de Heras Ciechomski, Shuji Yamashita, Akito Tabira, Takahiro Okuhara, Takeshi Kubojima, Yasunori Okada, Kotaro Oka, Robin Mange, Michael Unger, Akira Funahashi, Heinz Koeppl

**Affiliations:** 1 Department of BioSciences and Informatics, Keio University, Yokohama, Kanagawa, Japan; 2 Automatic Control Laboratory, Swiss Federal Institute of Technology, Zurich, Switzerland; 3, ScienceVisuals Sarl, Lausanne, Switzerland; 4 Department of Pathology,School of Medicine, Keio University, Tokyo, Shinjuku-ku, Japan

## Abstract

In our previous study, we introduced a combination methodology of Fluorescence Correlation Spectroscopy (FCS) and Transmission Electron Microscopy (TEM), which is powerful to investigate the effect of intracellular environment to biochemical reaction processes. Now, we developed a reconstruction method of realistic simulation spaces based on our TEM images. Interactive raytracing visualization of this space allows the perception of the overall 3D structure, which is not directly accessible from 2D TEM images. Simulation results show that the diffusion in such generated structures strongly depends on image post-processing. Frayed structures corresponding to noisy images hinder the diffusion much stronger than smooth surfaces from denoised images. This means that the correct identification of noise or structure is significant to reconstruct appropriate reaction environment *in silico* in order to estimate realistic behaviors of reactants *in vivo*. Static structures lead to anomalous diffusion due to the partial confinement. In contrast, mobile crowding agents do not lead to anomalous diffusion at moderate crowding levels. By varying the mobility of these non-reactive obstacles (NRO), we estimated the relationship between NRO diffusion coefficient (*D*_nro_) and the anomaly in the tracer diffusion (*α*). For *D*_nro_=21.96 to 44.49 *μm*^2^/*s*, the simulation results match the anomaly obtained from FCS measurements. This range of the diffusion coefficient from simulations is compatible with the range of the diffusion coefficient of structural proteins in the cytoplasm. In addition, we investigated the relationship between the radius of NRO and anomalous diffusion coefficient of tracers by the comparison between different simulations. The radius of NRO has to be 58 nm when the polymer moves with the same diffusion speed as a reactant, which is close to the radius of functional protein complexes in a cell.

## Introduction

The complex physical structure of the cytoplasm has been a long-standing topic of interest [1,2]. The physiological environment of intracellular biochemical reactants is not one of well diluted, homogeneous space. This fact is in contradiction with the basic assumption underlying the standard theories for reaction kinetics [3]. The difference may render actual *in vivo* reaction processes deviate from those *in vitro* or *in silico*. Lately, we showed the results of a combined investigation of Fluorescence Correlation Spectroscopy (FCS) and Transmission Electron Microscopy (TEM) [4,5]. We examined the effects of intracellular crowding and inhomogeneity on the mode of reactions *in vivo* by calculating the spectral dimension (*d*_*s*_) which can be translated into the reaction rate function. We compared estimates of the anomaly parameter, obtained from FCS data, with the fractal dimension from an analysis with transmission electron microscopy images. Therefrom we estimated a value of *d*_*s*_=1.34±0.27. This result suggests that the *in vivo* reactions run faster at initial times when compared to the reactions in a homogeneous space. The result is compatible with the result of our Monte Carlo simulation. Also, in our further investigation, we confirmed by the simulation that the above-mentioned *in vivo* like properties are different from those of homogeneously concentrated environments. Also other simulation results indicated that the crowding level of an environment affects the diffusion and reaction rate of reactants [6-9]. Such knowledge of the spatial condition enables us to construct realistic models for *in vivo* diffusion and reaction systems.

The novel points of this study are the following three: 

(i) we investigated the influence of the mobility of non-reactive obstacles (NRO) on the anomaly coefficient,

(ii) we investigated the influence of the size of the NROs, and

(iii) we reconstructed the static simulation space based on TEM images and run diffusion tests in these virtual volumes as well

in order to make the *in silico* simulation environment more realistic. The *in vivo* NROs have a wide size distribution and complex shapes. Based on our simulations we can suggest simpler systems with just one class of NROs which result in the same properties in the observed effective diffusion of the tracer molecules in the complex environment and experimental results.

While several projects investigated diffusion and reaction within compartments like the ER [10,11], this study aims at resolving the diffusion and reaction of cytosolic proteins outside of these structures, for instance signaling molecules that have to travel from the plasma membrane to the nucleus [12,13]. Cryoelectron tomography can be used to obtain a 3D reconstruction of only the scanned cell section [14,15]. Statistical methods, in contrast, can be used to learn the properties of the 3D space and to generate many samples from it [16,17]. In order to generate reaction volumes with the same properties like the TEM images, we therefore learned the image statistics. This enables us to test the influence of the structures such as mitochondria and membrane enclosed compartments on the diffusion and reaction of molecules in the cytosol. By using state-of-the-art volume visualization techniques we can also show the shape of the generated volumes.

The generated structures are used for a volumetric 3D pixel (voxel)-driven graphical representation, which was further filtered into a smooth analytic surface using the software package BioInspire [18,19]. This analytic conversion for the visualization was done to better understand the properties of the 3D structure, which is not obvious from single 2D slices. The analytic surface is also the natural description of large intracellular objects like membrane enclosed compartments or mitochondria [11,16] and avoids the discreteness of pixel/voxel-based approaches [20]. The 3D ray tracing visualization package BioInspire is used to interactively sample the analytical surface to create the final image; therefore, never losing any details by going over some intermediate representation such as a triangle mesh as is common in literature [21,22].

Generally, TEM images visualize the information of scattering/absorption or permeation of electron rays through a sample slice of the cell. The electron rays are detected by charge-coupled devices and converted to grey scale images. The part in a sample section where electrons have been scattered or absorbed appear darker on the image, while the parts permeating electron rays appear white. There exist many imaging studies which investigated intracellular structures by electron microscopy. In those images, organelle, such as nucleus, mitochondria, rough endoplasmic reticulum, zymogen granules, Golgi complex, etc., appear as clear shadows, resulting from scattered or absorbed electron rays.

Based on the above reasons, we assumed that the black segment in the TEM images consisted of solid structures comprising the non-reactive obstacle. Simultaneously, the non-reactive surface can provide anchorage for small mobile molecules. The faint segment areas in TEM images presumed to be made up of sol proteins, which formed the main reaction chamber for the intracellular reactants.

Besides the (at least temporarily) static structures the cytoplasm is known to be filled with all kinds of mobile-crowding molecules [2]. Therefore, we added the mobility of the NRO and their size to the parameters that are investigated in this study.

In our former simulation, we used just one size of NRO, which could, e.g., represent single molecular obstacles [4,5]. But in a cell, many of those molecules representing the NRO exist as complexes or polymers, for instance cytoskeletal proteins. In order to include this information, we analyzed if the overall radius of the obstacles would affect the diffusion and reaction processes. Especially, we checked the results obtained in such simulations for anomalous diffusion, which is a sensitive probe for crowding conditions [9].

Anomalous diffusion is a common phenomenon in cell biology [23] but was previously defined by using a random walker on percolation clusters [24]. Percolation theory deals with the number and properties of clusters which are formed as follows [25]; each site of a very large lattice is occupied randomly with probability *p*, independent of its neighbors. The resulting network structure is the target of percolation theory [26]. When the probability *p* is over the critical value (*p*_*c*_), the cluster reaches from one side to the opposite side of the lattice. This *p*_*c*_ is the threshold to undergo phase transition like the gelation of polymer sol. Anomalous diffusion is observed when the reaction space is occupied inhomogeneously with obstacles until the relative volume of obstacles reaches close to the threshold. The value of *p*_*c*_for the 3D cube is 0.312 [27].

In several numerical simulations including our model, a percolation lattice is used as a simple example of the disordered medium [7,28,29] and we found that it is similar to the *in vivo* reaction space. Likewise the structured *in vivo* reaction space is similar to porous media [6,30]. Such structures, which are often self-similar, can readily be seen under the TEM and are easily generated for instance by self-organizing molecules such as titanium dioxide and sol–gel powders.

When *p*=1, the cluster becomes a regular lattice without disorder. If the non-obstructed space in the cell forms such a regular lattice, the time dependency of the mean squared displacement (MSD) of a random walker on the lattice grows linear with time. On the other hand, if the random walker is confined at a specific volume, the MSD converges to a constant [31]. The case between these two extreme cases was named anomalous diffusion by Gefen et al. [24]. The exponent *α*represents the anomaly of the MSD [23]:

(1) $$\langle {\left(\vec{x}(t)-\vec{x}(0)\right)}^{2}\rangle =\Gamma t^{\alpha }$$

We estimated diffusion constants of NRO based on simulation results in different environments. Our *in silico* models enables us to verify the consistency of the hypothesis that the intracellular component is built using a self-organization and that the structure provides a percolation cluster-like environment for soluble molecules. We computed *α*from the Monte Carlo simulations in these virtual environments, as well as *D*(*t*), and compared it with the experimental results from FCS measurements to find the parameters of the *in silico* models which match the *in vivo* results.

## Main text

### Reconstruction of reaction space based on TEM image data

**Figure 1:**
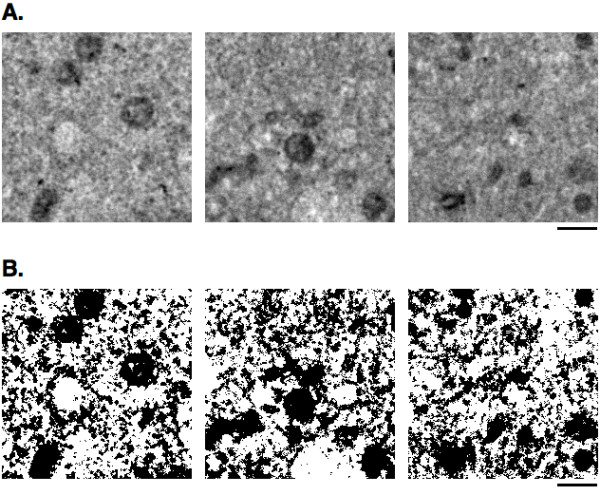
**Material TEM images. (A)** Original TEM images of the cytoplasmic region of 3Y1 cell for reaction space reconstruction.These images were captured by 1,000 magnifications. bar=1.0 *μ*m=56.8 pixels. **(B)** The binarized images of the photos **(A)**. The binarizing algorithm is described in “Methods”.

**Figure 2:**
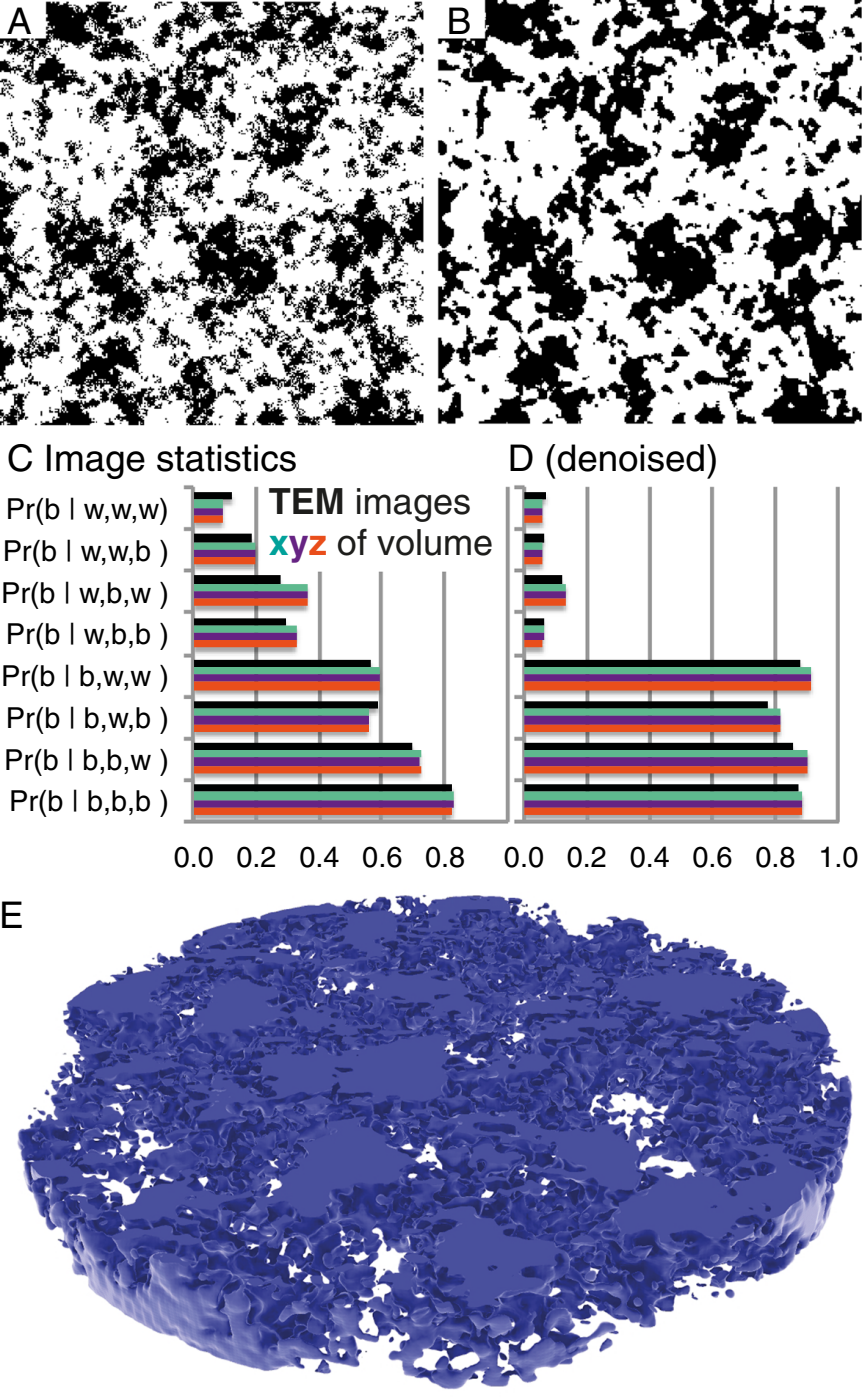
**Reconstructed reaction space based on TEM images for the reaction space. (A, B)** Sample images from the generated 3D space. **(C, D)** Comparison of original TEM image statistics and generated volume statistics. The reconstructed space has 17.6×17.6×17.6 nm resolution. **(B, D)** Low pass filtered by a median filter in order to reduce noise. **(E)** Visualization of the 3D structure by raytracing. See SI movie for a complete overview of the 3D reaction space.

**Figure 3:**
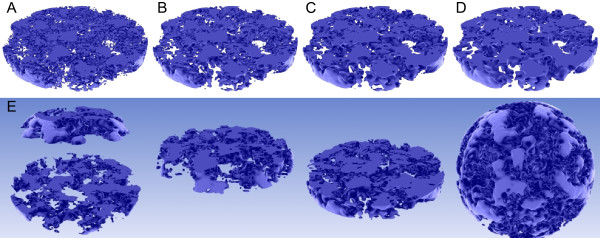
**Surface generation of the NRO structure.** Filtered versions of the above images going from left to right (**A**: 0%, **B**: 15%, **C**: 25%, and **D** 50% of the original voxel resolution) require (1.26, 1.21, 1.07, and 0.89 GB) of memory with an initial memory footprint of 0.49 GB, which amounts to around 50 MB per 1 million voxels. This reflects a linear memory usage with predictable performance requirements as the number of input voxels grow. Depending on the number of control points and coarse graining of the data points the surface becomes smoother, thus improving the perception of the overall 3D structure. The excluded volume grows slightly with the coarse graining and at the high value of D too many details of the structure are lost. **(E)** different slices of the reaction volume. The complete volume is also shown in the SI video (Additional file 1).

Based on TEM images (Figure 1) the intracellular environment was reconstructed (Figures 2 and 3) as described in Methods,“Generation of virtual cellular structures”. The 3D visualization of the static NRO structure helps to grasp the properties of the volume, which cannot be seen from single 2D images. A video showing the complete volume and sweeping through it is available as Supporting material (see Additional file 1).

The 1D statistics about neighboring pixels/voxels is sufficient to generate similar structures in two and three dimension applying an isotropy assumption. The structures show a wide size distribution in 2D images and a tubular network in the 3D volume. Only completely spherical structures are not generated in the present approach. The applied filters in the volume generation process have a tendency to increase the size of structures (eroding) or to reduce it (dilation). By controlling the NRO volume fraction in the process we could create volumes which have the same NRO volume fraction like the TEM images. Note that the smoothing of the surface for visualization likewise can increase the volume occupied by NROs (cf. Figure 3).

With respect to the diffusion of molecules through such structures the identification of the true fine-grained structure becomes very important. The diffusion test simulations in these 3D structures were performed with the continuous space discrete time Brownian dynamics simulation [6,32,33] (see Methods “Diffusion simulations in the virtual environment”). In the rather noisy structure corresponding to the thresholded TEM images, the diffusion is hindered much stronger than in a smoothed structure. We fitted the observed MSD to Equation (1) yielding *Γ*=3.37±0.14 in the noisy volume and *Γ*=3.79±0.15 in the smooth volume, i.e., the MSD grows faster in the smooth volume. The anomaly is *α*=0.940±0.004 and *α*=0.948±0.005, respectively. All simulations stopped, when the first of the 10,000 molecules starting from the center had reached the surface of our test volume—which restricts a further increase of the MSD. This time span/distance is not sufficient to leave the anomalous regime. The effective diffusion coefficient is on average reduced to 63% of the input value in the noisy volume and to 70% in the smooth volume at this point in time. Especially, the larger surface of the noisy volume leads to an increase in the excluded volume for finite particle radii, which is consistent with an increased reduction of the diffusion. Therefore, the more fragmented space leads to a stronger reduction in the diffusion [6].

**Figure 4:**
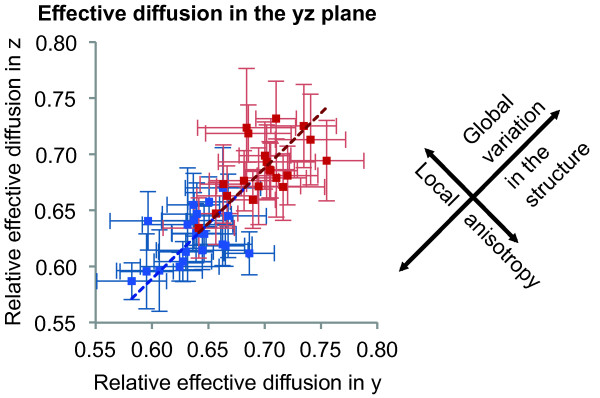
**Isotropy of the effective diffusion in the virtual cytoplasm.** Local anisotropy and global variation in the observed diffusion in different structures.

Also depending on the local structure the effective diffusion varies. As indicated in Figure 4, the structures can (locally) vary in their isotropy, leading to an anisotropic diffusion. It is especially important that the reaction space reconstruction process leads to isotropic structures because even slight deviations are sensitively recognized by the diffusion process. Likewise the original microscope data where each voxel is 17.6×17.6×60 nm are non-isotropic.

The comparison of the diffusion properties in the reconstructed reaction space and FCS measurements shows that the static (or at least temporarily static) structures are not sufficient to explain *in vivo* diffusion. The anomaly coefficient *α*=0.94 does not match the values observed in *in vivo* FCS measurements (*α*=0.768±0.14) [4,5]. Especially, the molecular crowding by mobile NROs seems to have an important effect [9,34]. The computational complexity of the multitude of interactions between all particles and the dimension of the simulation-parameter space however renders the analysis within such a detailed 3D volume structure impossible. Therefore, we investigated the influence of mobile NROs within a scalable discrete lattice-based simulation framework.

### Dynamics of NRO change the diffusion and reaction speed

We performed Monte Carlo simulation with mobile NRO in our lattice-based simulation space described in Methods “Lattice-based Monte Carlo simulation” (the lattice-based simulator is also included as Additional file 2 and available from [35]). The motivation to move the NRO despite the increased computational complexity is to make the simulation environment compatible with realistic intracellular conditions, and to investigate if we can find a simulation-parameter regime matching our former FCS results [4,5].

**Figure 5:**
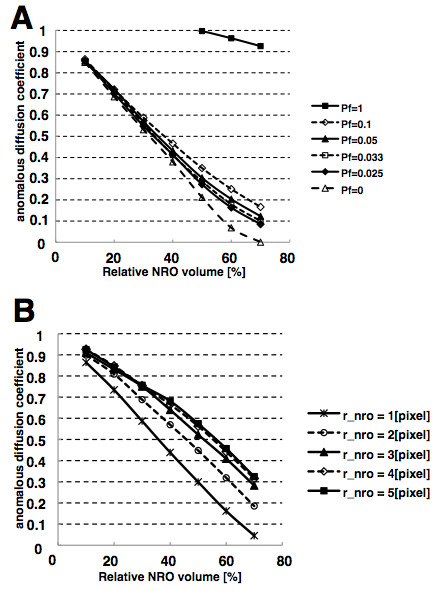
**Monte Carlo simulation with varying value of mobility and aggregation level of NRO. (A)** If all NRO are mobile with the same speed as the reactant and have the same size like it, the reactants in a cell show nearly a normal diffusion independent from the level of crowdedness. If the mobility of NRO is lower than the reactant diffusion in the reaction space, the reactants show anomalous diffusion as observed by FCS experiments. The different lines show the results produced by the different levels of crowding (0–70% of the volume is occupied by NRO). **(B)** Mobility of NRO is the same as the mobility of the reactant while the size is varied.

First, if the jump probability describing the mobility of the particles (*P*_*f*_) of the reactants equals the jump probability of the NROs (i.e., *P*_*f*_=1), the diffusion of reactants was independent from the crowding level of their environment. They show normal diffusion instead of anomalous diffusion (Figure 5A). By FCS analyses, we observed anomalous diffusion of green fluorescent protein (GFP) in cytoplasm. The simulation results with the NRO jump probability *P*_*f*_=1 thus was not compatible with experimental results. Especially, when the relative volume of NROs is lower than 50%, the diffusion of the reactants shows no anomalous subdiffusive behavior.

Starting from this incompatibility with the experimental results, we varied the following two parameters: (i) the probability which determines the mobility of NRO in the simulation space and (ii) the radius of NROs to analyze the effect of the size of NROs on the diffusion of the reactants.

### NRO mobility which leads to matching diffusion with experimental results

We varied the jump probability *P*_*f*_, which determines the mobility of NRO in the simulation space (Figure 5B). In this analysis, we fixed the size of NRO to occupy only one lattice site (i.e., single or small crowding molecules). The frequency of NRO movement was given in the range from 1/40 to 1/10 of the frequency of reactant moves, which move in every simulation step. This means that the NRO move once per 10 steps (*P*_*f*_=1/10), once per 20 steps (*P*_*f*_=1/20), once per 30 steps (*P*_*f*_=1/30), once per 40 steps (*P*_*f*_=1/40), or never (*P*_*f*_ = 0), respectively.

The results in Figure 5A show that if *P*_*f*_is less than 1/10, diffusing reactants show the anomalous subdiffusive behavior for all tested NRO levels from 10 to 70%. This result is in agreement with previous works which indicated that the more static NROs result in a stronger confinement of the reactants [6,31], hence a more anomalous behavior (smaller *α*).

For all *P*_*f*_<1, we can obtain an anomalous parameter compatible with our experimental results (*α*=0.768±0.14) with about 20% relative volume of NRO in the reaction space. The estimated *P*_*f*_ value to reproduce the compatible *α*is 0.2383 to 0.3689. This means that the reactants move 2 to 5 times faster than the NROs in the reaction volume. However, the estimated relative volume amount is less than the occupied volume in the TEM images of 37%. Previous studies showed that the NRO-effect on the diffusion strongly depends on the size of the NROs [6,36]. Therefore, also the size has to be taken into account.

### NRO size which leads to matching diffusion with experimental results

We also varied the aggregation level of NRO in the simulation space (Figure 5B). In this analysis, we fixed the mobility of the NROs to the same rate like the mobility of the reactants (*P*_*f*_=1).

The radius of the NRO was varied from 1 to 5 pixels. The original size (*r*_nro_=1) means that the object occupies 8 pixels. We assumed the reactants diffuse in cytoplasm. Because the reactants affect the moves of the NROs in the same way like the NROs block the way of the reactants, the concentrations of both NROs and reactants have to set in the right proportion. In order to adopt our simulation environment to the case of cytoplasmic enzyme, we chose 1.0 *μ*M as the approximate concentration of the reactant. Our simulation environment for varying NRO radius is 1000 reactants in the lattice with 50×50×50 total sites. To reconstruct the realistic intracellular environment by our simulation space, we assume the size of 1 pixel equals to 77.8 nm. This is about 15 times larger than the diameter of GFP, which is the molecule for which we analyzed the diffusion in a cytoplasmic region. Also, the approximate compartment size is 64 *μ*m^3^ = 64 fl. This volume is acceptable as a part of cytoplasm; the expected whole volume of cytoplasm of a cell is 2.8 pl [37]. Now the radius of NRO varied from 1 to 5 pixels means the diameter of NRO is 155.6 to 778 nm.

By changing the size of NRO, we find that the relative NRO volume is different for each different NRO size to produce compatible anomalous diffusion coefficient with experimental results. When the NRO size is small (155.6 nm, i.e., 30 times larger than a reactant), a cell can involve only 15 to less than 20% relative volume of NRO to produce a compatible anomalous diffusion coefficient with experimental results. If the NRO size is large (778 nm, i.e., 150 times larger than a reactant), a cell can involve over 30% relative volume of NRO to produce a compatible anomalous diffusion coefficient. This result is also consistent with a previous studies which showed that smaller objects have a much bigger influence on the diffusion of test molecules [6,36].

### Empiric relationship between ***α***, ***D***_**nro**_, and ***r***_**nro**_

We fitted the empiric functions given in Table 1 to the results of our Monte Carlo simulation with various conditions in order to find parameter ranges which are consistent with the results from FCS measurements. Note that these empiric functions do not need to have a physical meaning, but for instance show that the Stokes–Einstein relation D∝1/r is not valid in the cytoplasm, because due to the microscopic structure different radii exhibit different viscosity. For instance large molecules sense a bigger hindrance in their mobility and can even be trapped by the meshes of the cytoskeleton [2,6].

**Table 1:** **Empiric relations between** ***α*,** ***D***_**nro**_**, and** ***r***_**nro**_

	
Relationship between *α* and *D*_nro_	*α*=0.0093*D*_nro_ + 0.4606
Relationship between *α* and *r*_nro_	$\alpha =0.1302\times \ln r_{\text{nro}}+0.0976$
Relationship between *D*_nro_ and *r*_nro_	$D_{\text{nro}}=14.0\ln r_{\text{nro}}-39.0$

The empiric relations are fitted to the simulation results. We used the value $D_{\text{GFP}}=82\pm 2\mu {\text{m}}^{2}/\text{s}$ for GFP and its mutant protein in solution [38]. The last relation is then deduced from the first two for ***P***_***f***_**=1.0**and a NRO volume fraction of 37%.

The relation between *D*_nro_and *r*_nro_(Table 1, third equation) is calculated from the first two equations in Table 1 for the condition *P*_*f*_=1. Based on the appropriate size of the NRO from the previous section and the relationship with *r*_nro_ we conclude that *D*_nro_=21.96 to 44.49 *μ*m^2^/s in order to obtain the desired *α*in the simulation at the target NRO fraction of 37%.

This diffusion coefficient is still in the same range like the diffusion coefficient of GFP in cytoplasm. On the one hand it is rather fast for large molecules but on the other hand our model *in silico* cytoplasm is just constructed out of one class of NROs compared to the complex size distribution *in vivo*[9,34]. The diffusion coefficient is not more than 10 times faster than the diffusion coefficient of large macromolecules (e.g., microtubule) in cytoplasm, thus supporting that our results are in a realistic physiological regime.

On the other hand, if the diffusion of NRO occurs at the physiological macromolecule level (ex. tubulin in cytoplasm is measured as 4–10 *μ*m^2^/s [39]), the diameter of NRO must be about 33–43 nm. This is smaller than the single NRO in our simulation. That means if the reaction space is crowded only with this size of obstacles, the anomalous diffusion constant will be smaller than the physiological value at the relative NRO volume fraction of 37%, which we found in our TEM image data. This value of relative NRO volume should be independent from the mobility state of the NROs.

## Conclusions

We can conclude from simulation results in the reconstructed reaction space that the correct identification of noise or concrete structures in TEM images is very important because the diffusion strongly depends on it. The reconstructed tubular structures are consistent with, e.g., ER structures [11]. The structures are static in simulations of that reconstructed space (at least on the short timescales of the simulation), but future work aims at modeling the spatial dynamics of such membrane enclosed compartments [40]. The present generated structures could serve as a starting point for the size distribution of the compartments. Finally, a detailed and multi-scale simulation should include both the quasi-static cellular structures and the mobile NROs responsible for the majority of the molecular crowding effects. At the same time, investigation of the mixing ratio of differently sized NROs is also necessary in order to find a functional size distribution.

As the microscope data are discretizing the cell internal structures one could argue that the simulation should also use the 3D analytical surface representation, reconstructed inside the BioInspire visualization software. At the moment the simulation is not using this surface as the interfaces between the simulation and visualization are currently being defined. For an investigation of transient anomalous diffusion in such structures [23], much longer time spans need to be covered, which means that particles will diffuse much further away. Therefore, periodic boundary conditions for the volume are necessary. The reaction space might also be reconstructed based on the Fourier transform of the TEM images, which would lead to smooth boundaries under periodic boundary conditions.

The TEM image-reconstruction for a realistic simulation space gave us (i) an impression how the microscopic intracellular environment is structured in 3D and (ii) lets us further compare the results with that of lattice based and more scalable simulations, which also includes mobile NROs. By searching a compatible condition between the results of TEM-reconstructed space and artificial space, we could estimate the parameters for *in silico* simulation environments with realistic intracellular structures and dynamics.

Due to computational limitations these environments have to be tremendously simplified compared to the complexity of the *in vivo* system. Thus, our efforts match for instance the approach of Hou et al. [41] trying to create a simplified yet realistic *in vitro* model of the cytoplasm.

We confirmed that the diffusion characteristics of inert test molecules in a crowded space are preserved in the characteristics of molecules which take part in a Michaelis-Menten reaction by using discrete reaction space [42]. The reaction proceeds quickly at the beginning, but later on the reactants are exhausted slowly in our simulations. This result may mean that the intracellular environment transforms reaction processes in a cell from the *in vitro* reaction in a fractal manner [8]. It is comparable to the classic mass action system with a time-dependent rate constant. Also the observable effective reaction rate constant depends on the level of crowding and the effective diffusion, and might sensitively react in the case of anomalous diffusion [32]. These results support the importance to confirm detailed structures of the reaction space because the reaction environment affects the reaction process.

Therefore, the next challenge for *in vivo* oriented simulations will be performing simulations of bimolecular enzymatic reaction processes in the reconstructed reaction volume based on true cell environment, also by estimating the concrete value of environmental dynamics, and possibly by mixing static structures and mobile NROs.

## Methods

### Cell culture

Cell culture reagents for 3Y1 cells were obtained from Wako Pure Chemical Industries, Ltd. (Japan). The cell lines were routinely cultured in Dulbecco’s Minimal Essential Medium supplemented with 10% fetal bovine serum in a 5% CO_2_ incubator. We obtained 3Y1 cell line from Japanese Collection of Research Bioresources (JCRB) Cell Bank for use at Keio University.

### Transmission electron microscopy

We obtained 101 images of rat fibroblast 3Y1 cells. We selected those images from the cytoplasmic regions, mainly at a magnification 1000.

The cells were collected on the day when the cells reached at the confluent condition in order to obtain a homogeneous population in their cell cycle (G1 to G0 cells).

In preparation for TEM, the cells were fixed with 4% formaldehyde and 2% glutaraldehyde in 0.1-M phosphate buffer (pH 7.4) for 16 h at 4°C, and successively with 1% osmium tetraoxide in 0.1-M phosphate buffer (pH 7.4). The cells were dehydrated in graded ethanol and embedded in epoxy resin. Ultrathin sections (approximately 60-nm thick) were prepared with a diamond knife and were electron-stained with uranyl acetate and lead citrate, and were examined using an electron microscope (H-7650; Hitachi Ltd.).

First, the TEM images were binarized into objects and background using the auto-thresholding function of ImageJ (http://rsbweb.nih.gov/ij/; see Figure 1). Briefly, this algorithm computes the average intensity of the pixels at below or above, a particular threshold. It then computes the average of these two values, increments the threshold, and iterates the process until the threshold is larger than the composite average. That is,

(2) $$\mathrm{threshold}=\frac{\left(\text{average background}+\text{average objects}\right)}{2}.$$

Subsequently, the binary images were translated into a 1-0 matrix in Matlab to reconstruct the simulation space. The simulation space for Figures 2, 3, and 4 was reconstructed based on TEM images as indicated below.

### Generation of virtual cellular structures

In order to reconstruct the intracellular environment we learned the following statistics from the thresholded binary TEM images (cf. Figure 1B): *P*_*b*_(*I*(*px*_*i*_)=1|*I*(*px*_*i*−1_),*I*(*px*_*i*−2_),*I*(*px*_*i*−3_)), the probability that this pixel *px*_*i*_is black (*I*(*px*_*i*_)=1), given the sequence of the neighboring three pixels, averaged over all directions (cf. Figure 2C). Likewise, we learned the probability of a pixel being black which is between two other pixels (separated by a distance *j*), and the average blackness (0.3755).

The 300×300×300 px *in silico* volume is generated by drawing lines from *P*_*b*_, each separated by 16 px in all directions. Next, we interpolated the pixels in between the lines (distance 8, 4, 2, and 1 px) to generate the complete volume. The generated volume is then iteratively processed by filtering it (erosion and dilation) until its ${\hat{P}}_{b}$ in all directions equals the empirical *P*_*b*_of the images (cf. Figure 2A,C). In order to preserve not only big structures but also finer objects in the processed volume, the raw volume was fed back into the processed volume repeatedly by averaging over both images, while the weight of the raw image was reduced in each iteration. In order to produce a smoother surface, the volume was also low pass filtered (cf. Figure 2A–D). The necessary 3D filters were created based on ordfilt3 by Olivier Salvado from the Matlab central File Exchange (File ID: #5722). The present Matlab code to generate the volumes is available as Additional file 3.

In order to avoid boundary effects only the pixels 10-290 are used subsequently in the simulations, and accordingly a sphere with a diameter of 4.928*μ*m is created at the scale of 1 px = 17.6 nm.

### Visualization

The 3D NRO structure described in the previous section—even if filtered twice, once in 2D with ImageJ (section “Transmission electron microscopy”) and once in 3D in Matlab (cf. Figure 2A,B)—still contains high-frequency components from image noise and the discretization of data into voxels. Image stacks acquired from TEM are discretizations of the actual natural analytic (or at least very highly detailed) environment of the cell’s internal structures, which is why the direct visualization of the voxel space itself only reveals the coarse grained, cubic 3D environment. As input to the BioInspire raytracing engine, a total of 12.5 million voxels (4.5 million of which are occupied by NROs) were given, corresponding to the spherical subvolume of the simulation space. As touched upon in the introduction a 3D filter of the software package BioInspire was used to create a smooth surface by averaging over the 3D structure. The difference in non-processed data and filtered data can be seen in Figure 3 where the number of control points and parameters is adjusted. Clearly, the filtered version with a smoother surface is preferable for a clear visualization of the 3D structure. A section of the volume is shown in Figure 2 for comparison with the 2D 300×300 pixel image of single slices.

### Diffusion simulations in the virtual environment

The continuous space discrete time diffusion simulator as described in [32] is used to simulate the diffusion of inert tracer molecules through a cell which contains the generated structures. The structures are represented by a binary 3D grid of spheres at the positions of black voxels of the generated volume. The static spheres had a radius of *r*_*s*_=10.92 nm, such that their volume matches the volume of each pixel of (17.6 nm)^3^. We performed the simulations in 20 different structures to average over the different realizations. The diffusion of tracer molecules with molecular radii of *r*_*i*_=2.6 nm was simulated with 10 sets of 1000 molecules each. All original diffusion coefficients are arbitrarily set to $D_{0}=1\mu {\text{m}}^{2}/s$, and *Δt* is chosen such that max*Δx*/(*r*_*i*_ + *r*_*s*_)=0.08, i.e. *Δt*=1.27×10^−7^*s*. The effective diffusion $D_{\mathrm{ef}\;f}=\langle {\left(x(t)-x(t_{0})\right)}^{2}\rangle /(2d(t-t_{0}))$ was obtained in 3 dimensions (*d*=3) as well as in each dimension separately (*d*=1). The test volume was a cell with a diameter of 4.928*μ*m and was accordingly filled with approximately 4.5 million obstacles. The simulations were performed on the Brutus computing cluster at ETH Zurich, needed 10 h for 0.15 s of physical time and 400 MB memory at max (non-parallelized, but the different sets were running in parallel). With a Intel Core i7 2600K at 3.5 GHz and 8 GB RAM 1×10^6^ steps, (i.e., 0.127s) of all 10000 particles of one set needed 3 h. The simulation is available from [33]. We used this virtual environment for the calculation of effective diffusion constant and for the investigation of the local anisotropy of the volume.

### Lattice-based Monte Carlo simulation

We also performed a scalable lattice-based Monte Carlo simulation and compared it with the results from the simulations in our virtual environment as well as experimental results from [4,5] by changing the size and mobility of NRO, in order to clarify the characteristics of such a crowded environment. This simulation is available from [35] or Additional file 2.

#### Diffusion simulation with immobile NRO

The simulation space is a 50×50×50 cubic lattice with periodic boundary conditions. The reaction space is randomly interspersed with NRO. The random walkers representing the diffusing reactants can jump to a neighboring lattice site in each iteration, which is selected randomly. If the chosen lattice site was previously empty, the reactant fills the site; if the site was occupied by an NRO, a new position is randomly allocated for the reactant. The simulator is implemented in the C++ programming language.

#### Reaction simulation with immobile NRO

The reaction simulated in our model is A+A→A. If the chosen lattice site of reactant *A*1 in a diffusion step is occupied by another reactant *A*2, *A*2 is obliterated and only *A*1 remains at the new lattice site.

#### Pseudo-mono reaction process simulation with mobile NRO

We changed the characteristics of NRO such that they can move randomly as well. Their probability to move *P*_*f*_ was varied from the same as reactants (*P*_*f*_=1) to 40 times smaller (*P*_*f*_=1/40), i.e., slower, to investigate the effect of NRO mobility to the reactants behaviors.

All NRO move as single independent molecules. The other conditions for this simulation remain the unchanged.

#### Pseudo-mono reaction process simulation with aggregated NRO

We also varied the diameter of NRO to test the effect of NRO size to the reactant behaviors. By this analysis, we investigated the condition relating with NRO aggregation level, which move with *P*_*f*_=1, i.e., with the same probability as the reactants. The other conditions for this simulation remain the unchanged.

## Additional files

## Competing interests

Dr.Pablo de Heras Ciechomski is the founder of ScienceVisuals, Sarl, which is developing products related to the research described in this article and developed through the Swiss Agency KTI for promotion of medical technologies. The terms of this arrangement have been reviewed and approved by the swiss Federal Institute of Technology, Zurich, Switzerland, in accordance with their respective conflict of interest policies.

## Supplementary Material

Addtional file 1 Video of the 3D volume. Dynamic exploration of the generated 3D virtual cytoplasm. Click here for file

Addtional file 2 Lattice-based simulator. Zip folder contains reaction–diffusion simulator with mobile and fixed lattice-based NROs of variable size. Code is written in C++ and requires the respective compilers. Click here for file

Addtional file 3 MATLAB code for volume generation. Zip folder contains original images to learn statistics from and MATLAB code to generate the 3D volumes. Requires MATLAB. Click here for file
