# Supplementary figures and images for: From microscopy data to in silico environments for in vivo-oriented simulations
Source: EURASIP J Bioinform Syst Biol. 2012 Jun 26;2012(1):7. doi: 10.1186/1687-4153-2012-7 (PMC3698665; doi:10.1186/1687-4153-2012-7)

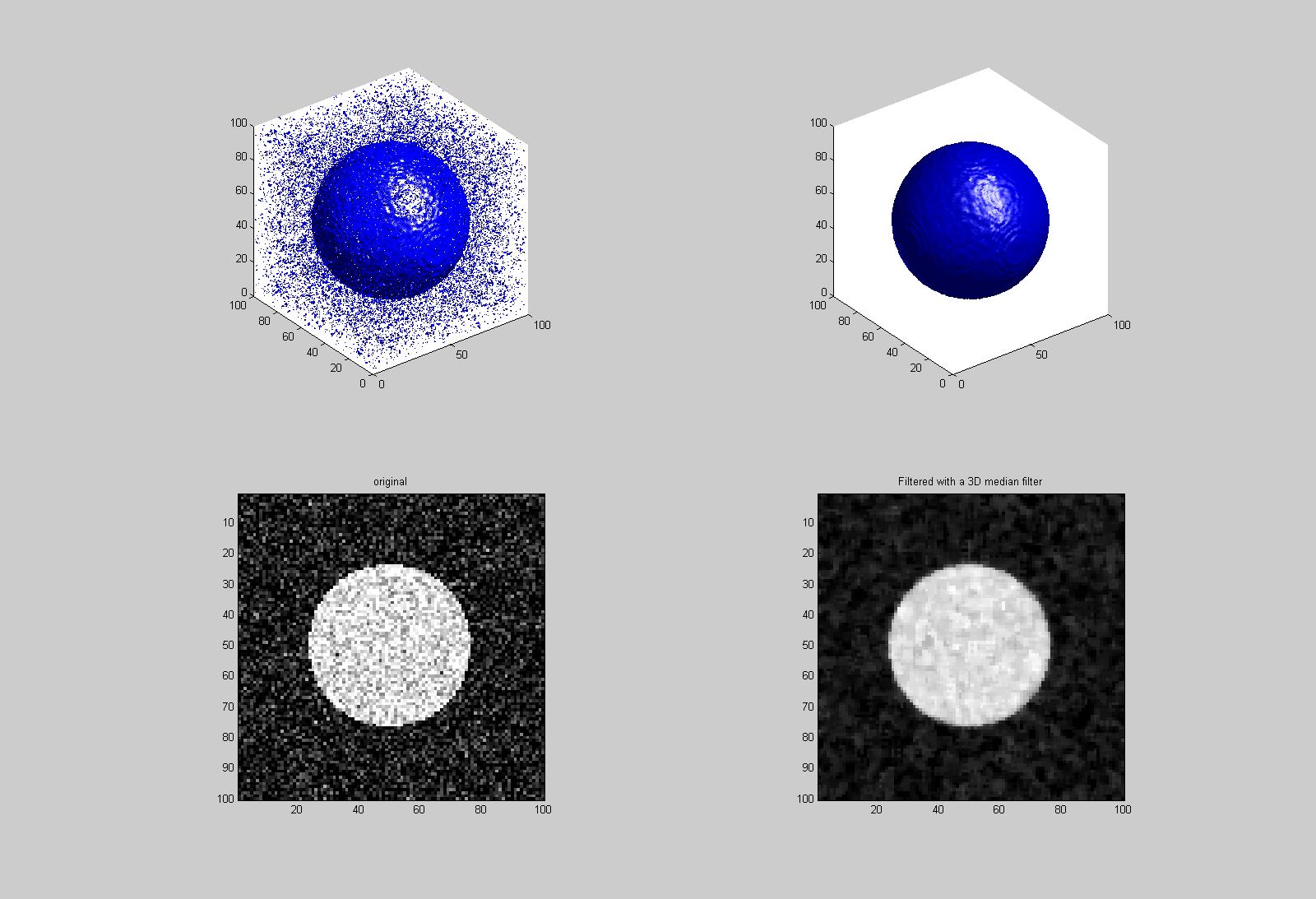

Supplement: Addtional file 3 — MATLAB code for volume generation. Zip folder contains original images to learn statistics from and MATLAB code to generate the 3D volumes. Requires MATLAB. [file 1687-4153-2012-7-S3.zip › Matlab_VolumeGenerator/SampleSet/ordfilt3/ordfilt3_picure.JPG]

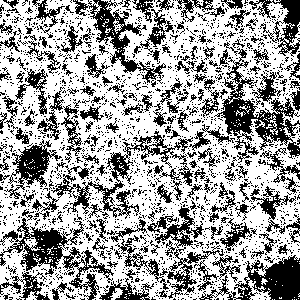

Supplement: Addtional file 3 — MATLAB code for volume generation. Zip folder contains original images to learn statistics from and MATLAB code to generate the 3D volumes. Requires MATLAB. [file 1687-4153-2012-7-S3.zip › Matlab_VolumeGenerator/SampleSet/original_figures/128binary.tif]

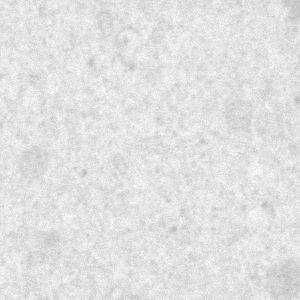

Supplement: Addtional file 3 — MATLAB code for volume generation. Zip folder contains original images to learn statistics from and MATLAB code to generate the 3D volumes. Requires MATLAB. [file 1687-4153-2012-7-S3.zip › Matlab_VolumeGenerator/SampleSet/original_figures/128original.tif]

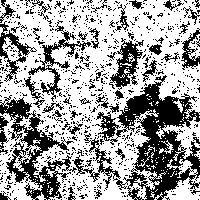

Supplement: Addtional file 3 — MATLAB code for volume generation. Zip folder contains original images to learn statistics from and MATLAB code to generate the 3D volumes. Requires MATLAB. [file 1687-4153-2012-7-S3.zip › Matlab_VolumeGenerator/SampleSet/original_figures/129binary.tif]

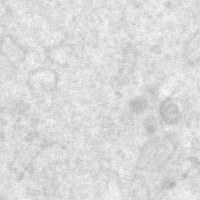

Supplement: Addtional file 3 — MATLAB code for volume generation. Zip folder contains original images to learn statistics from and MATLAB code to generate the 3D volumes. Requires MATLAB. [file 1687-4153-2012-7-S3.zip › Matlab_VolumeGenerator/SampleSet/original_figures/129original.tif]

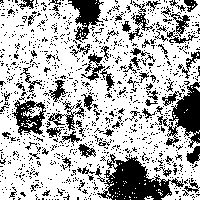

Supplement: Addtional file 3 — MATLAB code for volume generation. Zip folder contains original images to learn statistics from and MATLAB code to generate the 3D volumes. Requires MATLAB. [file 1687-4153-2012-7-S3.zip › Matlab_VolumeGenerator/SampleSet/original_figures/130binary.tif]

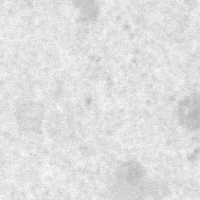

Supplement: Addtional file 3 — MATLAB code for volume generation. Zip folder contains original images to learn statistics from and MATLAB code to generate the 3D volumes. Requires MATLAB. [file 1687-4153-2012-7-S3.zip › Matlab_VolumeGenerator/SampleSet/original_figures/130original.tif]

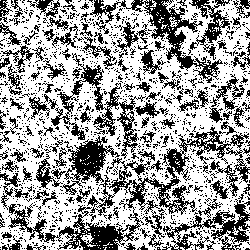

Supplement: Addtional file 3 — MATLAB code for volume generation. Zip folder contains original images to learn statistics from and MATLAB code to generate the 3D volumes. Requires MATLAB. [file 1687-4153-2012-7-S3.zip › Matlab_VolumeGenerator/SampleSet/original_figures/131binary.tif]

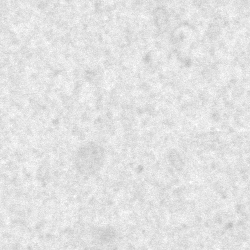

Supplement: Addtional file 3 — MATLAB code for volume generation. Zip folder contains original images to learn statistics from and MATLAB code to generate the 3D volumes. Requires MATLAB. [file 1687-4153-2012-7-S3.zip › Matlab_VolumeGenerator/SampleSet/original_figures/131original.tif]

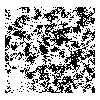

Supplement: Addtional file 3 — MATLAB code for volume generation. Zip folder contains original images to learn statistics from and MATLAB code to generate the 3D volumes. Requires MATLAB. [file 1687-4153-2012-7-S3.zip › Matlab_VolumeGenerator/SampleSet/original_figures/132binary.tif]

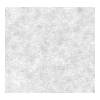

Supplement: Addtional file 3 — MATLAB code for volume generation. Zip folder contains original images to learn statistics from and MATLAB code to generate the 3D volumes. Requires MATLAB. [file 1687-4153-2012-7-S3.zip › Matlab_VolumeGenerator/SampleSet/original_figures/132original.tif]

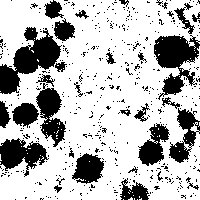

Supplement: Addtional file 3 — MATLAB code for volume generation. Zip folder contains original images to learn statistics from and MATLAB code to generate the 3D volumes. Requires MATLAB. [file 1687-4153-2012-7-S3.zip › Matlab_VolumeGenerator/SampleSet/original_figures/138binary.tif]

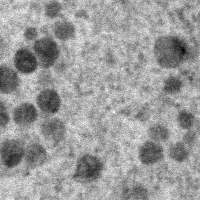

Supplement: Addtional file 3 — MATLAB code for volume generation. Zip folder contains original images to learn statistics from and MATLAB code to generate the 3D volumes. Requires MATLAB. [file 1687-4153-2012-7-S3.zip › Matlab_VolumeGenerator/SampleSet/original_figures/138original.tif]

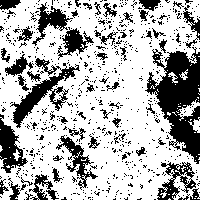

Supplement: Addtional file 3 — MATLAB code for volume generation. Zip folder contains original images to learn statistics from and MATLAB code to generate the 3D volumes. Requires MATLAB. [file 1687-4153-2012-7-S3.zip › Matlab_VolumeGenerator/SampleSet/original_figures/139binary.tif]

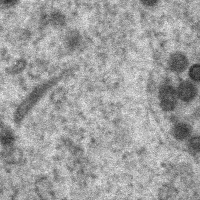

Supplement: Addtional file 3 — MATLAB code for volume generation. Zip folder contains original images to learn statistics from and MATLAB code to generate the 3D volumes. Requires MATLAB. [file 1687-4153-2012-7-S3.zip › Matlab_VolumeGenerator/SampleSet/original_figures/139original.tif]

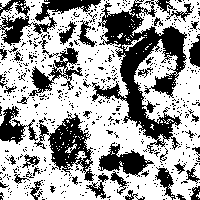

Supplement: Addtional file 3 — MATLAB code for volume generation. Zip folder contains original images to learn statistics from and MATLAB code to generate the 3D volumes. Requires MATLAB. [file 1687-4153-2012-7-S3.zip › Matlab_VolumeGenerator/SampleSet/original_figures/140binary.tif]

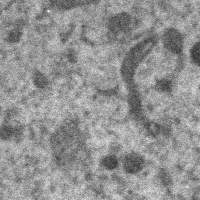

Supplement: Addtional file 3 — MATLAB code for volume generation. Zip folder contains original images to learn statistics from and MATLAB code to generate the 3D volumes. Requires MATLAB. [file 1687-4153-2012-7-S3.zip › Matlab_VolumeGenerator/SampleSet/original_figures/140original.tif]

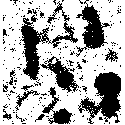

Supplement: Addtional file 3 — MATLAB code for volume generation. Zip folder contains original images to learn statistics from and MATLAB code to generate the 3D volumes. Requires MATLAB. [file 1687-4153-2012-7-S3.zip › Matlab_VolumeGenerator/SampleSet/original_figures/141binary.tif]

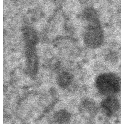

Supplement: Addtional file 3 — MATLAB code for volume generation. Zip folder contains original images to learn statistics from and MATLAB code to generate the 3D volumes. Requires MATLAB. [file 1687-4153-2012-7-S3.zip › Matlab_VolumeGenerator/SampleSet/original_figures/141original.tif]

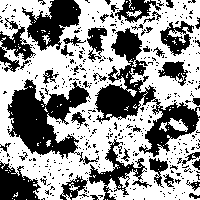

Supplement: Addtional file 3 — MATLAB code for volume generation. Zip folder contains original images to learn statistics from and MATLAB code to generate the 3D volumes. Requires MATLAB. [file 1687-4153-2012-7-S3.zip › Matlab_VolumeGenerator/SampleSet/original_figures/18binary.tif]

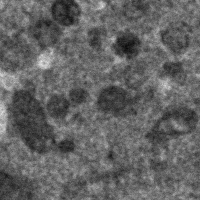

Supplement: Addtional file 3 — MATLAB code for volume generation. Zip folder contains original images to learn statistics from and MATLAB code to generate the 3D volumes. Requires MATLAB. [file 1687-4153-2012-7-S3.zip › Matlab_VolumeGenerator/SampleSet/original_figures/18original.tif]

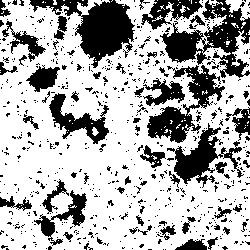

Supplement: Addtional file 3 — MATLAB code for volume generation. Zip folder contains original images to learn statistics from and MATLAB code to generate the 3D volumes. Requires MATLAB. [file 1687-4153-2012-7-S3.zip › Matlab_VolumeGenerator/SampleSet/original_figures/19binary.tif]

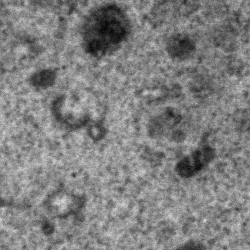

Supplement: Addtional file 3 — MATLAB code for volume generation. Zip folder contains original images to learn statistics from and MATLAB code to generate the 3D volumes. Requires MATLAB. [file 1687-4153-2012-7-S3.zip › Matlab_VolumeGenerator/SampleSet/original_figures/19original.tif]

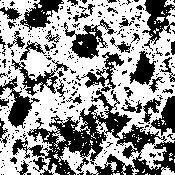

Supplement: Addtional file 3 — MATLAB code for volume generation. Zip folder contains original images to learn statistics from and MATLAB code to generate the 3D volumes. Requires MATLAB. [file 1687-4153-2012-7-S3.zip › Matlab_VolumeGenerator/SampleSet/original_figures/28binary.tif]

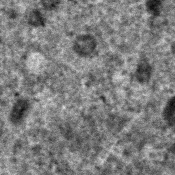

Supplement: Addtional file 3 — MATLAB code for volume generation. Zip folder contains original images to learn statistics from and MATLAB code to generate the 3D volumes. Requires MATLAB. [file 1687-4153-2012-7-S3.zip › Matlab_VolumeGenerator/SampleSet/original_figures/28original.tif]

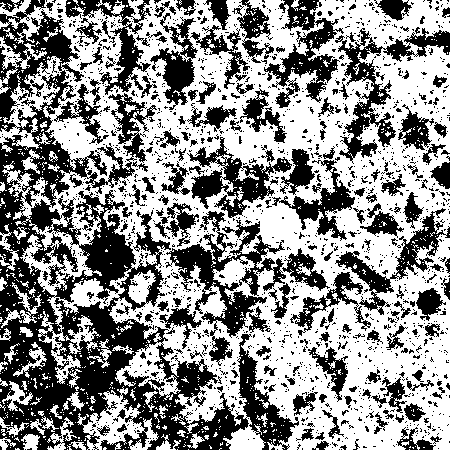

Supplement: Addtional file 3 — MATLAB code for volume generation. Zip folder contains original images to learn statistics from and MATLAB code to generate the 3D volumes. Requires MATLAB. [file 1687-4153-2012-7-S3.zip › Matlab_VolumeGenerator/SampleSet/original_figures/2binary.tif]

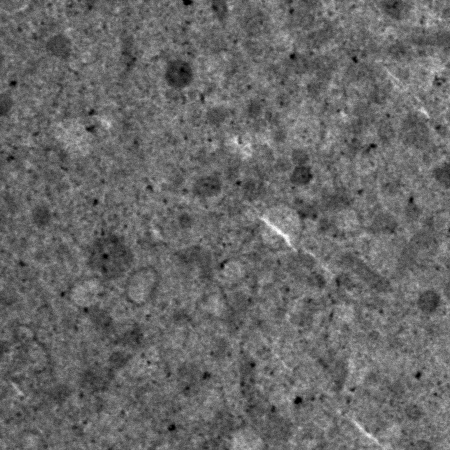

Supplement: Addtional file 3 — MATLAB code for volume generation. Zip folder contains original images to learn statistics from and MATLAB code to generate the 3D volumes. Requires MATLAB. [file 1687-4153-2012-7-S3.zip › Matlab_VolumeGenerator/SampleSet/original_figures/2original.tif]

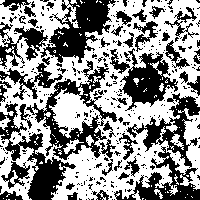

Supplement: Addtional file 3 — MATLAB code for volume generation. Zip folder contains original images to learn statistics from and MATLAB code to generate the 3D volumes. Requires MATLAB. [file 1687-4153-2012-7-S3.zip › Matlab_VolumeGenerator/SampleSet/original_figures/34binary.tif]

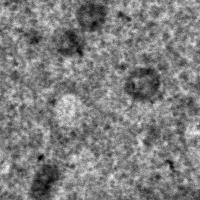

Supplement: Addtional file 3 — MATLAB code for volume generation. Zip folder contains original images to learn statistics from and MATLAB code to generate the 3D volumes. Requires MATLAB. [file 1687-4153-2012-7-S3.zip › Matlab_VolumeGenerator/SampleSet/original_figures/34original.tif]

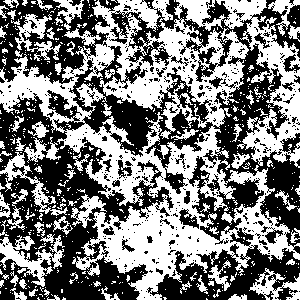

Supplement: Addtional file 3 — MATLAB code for volume generation. Zip folder contains original images to learn statistics from and MATLAB code to generate the 3D volumes. Requires MATLAB. [file 1687-4153-2012-7-S3.zip › Matlab_VolumeGenerator/SampleSet/original_figures/3binary.tif]

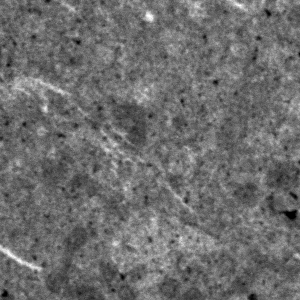

Supplement: Addtional file 3 — MATLAB code for volume generation. Zip folder contains original images to learn statistics from and MATLAB code to generate the 3D volumes. Requires MATLAB. [file 1687-4153-2012-7-S3.zip › Matlab_VolumeGenerator/SampleSet/original_figures/3original.tif]

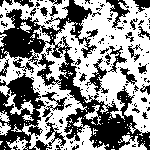

Supplement: Addtional file 3 — MATLAB code for volume generation. Zip folder contains original images to learn statistics from and MATLAB code to generate the 3D volumes. Requires MATLAB. [file 1687-4153-2012-7-S3.zip › Matlab_VolumeGenerator/SampleSet/original_figures/6binary.tif]

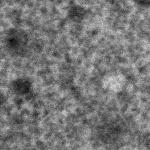

Supplement: Addtional file 3 — MATLAB code for volume generation. Zip folder contains original images to learn statistics from and MATLAB code to generate the 3D volumes. Requires MATLAB. [file 1687-4153-2012-7-S3.zip › Matlab_VolumeGenerator/SampleSet/original_figures/6original.tif]
